# A compromised specific humoral immune response against the SARS-CoV-2 receptor-binding domain is related to viral persistence and periodic shedding in the gastrointestinal tract

**DOI:** 10.1038/s41423-020-00550-2

**Published:** 2020-10-09

**Authors:** Fengyu Hu, Fengjuan Chen, Zhihua Ou, Qinghong Fan, Xinghua Tan, Yaping Wang, Yuejun Pan, Bixia Ke, Linghua Li, Yujuan Guan, Xiaoneng Mo, Jian Wang, Jinlin Wang, Chun Luo, Xueliang Wen, Min Li, Peidi Ren, Changwen Ke, Junhua Li, Chunliang Lei, Xiaoping Tang, Feng Li

**Affiliations:** 1 Guangzhou Eighth People’s Hospital, Guangzhou, 510440 China; 2 Shenzhen Key Laboratory of Unknown Pathogen Identification, BGI-Shenzhen, Shenzhen, 518083 China; 3 Guangdong Provincial Center for Disease Control and Prevention, Guangzhou, 511430 China; 4 BGI Education Center, University of Chinese Academy of Sciences, Shenzhen, 518083 China; 5 School of Biology and Biological Engineering, South China University of Technology, Guangzhou, 510006 China

**Keywords:** SARS-CoV-2, protective antibody, gastrointestinal infection, virus recurrence, Viral infection, Predictive markers

## Abstract

Severe acute respiratory syndrome coronavirus 2 (SARS-CoV-2) has been redetected after discharge in some coronavirus disease 2019 (COVID-19) patients. The reason for the recurrent positivity of the test and the potential public health concern due to this occurrence are still unknown. Here, we analyzed the viral data and clinical manifestations of 289 domestic Chinese COVID-19 patients and found that 21 individuals (7.3%) were readmitted for hospitalization after detection of SARS-CoV-2 after discharge. First, we experimentally confirmed that the virus was involved in the initial infection and was not a secondary infection. In positive retests, the virus was usually found in anal samples (15 of 21, 71.4%). Through analysis of the intracellular viral subgenomic messenger RNA (sgmRNA), we verified that positive retest patients had active viral replication in their gastrointestinal tracts (3 of 16 patients, 18.7%) but not in their respiratory tracts. Then, we found that viral persistence was not associated with high viral titers, delayed viral clearance, old age, or more severe clinical symptoms during the first hospitalization. In contrast, viral rebound was associated with significantly lower levels of and slower generation of viral receptor-binding domain (RBD)-specific IgA and IgG antibodies. Our study demonstrated that the positive retest patients failed to create a robust protective humoral immune response, which might result in SARS-CoV-2 persistence in the gastrointestinal tract and possibly in active viral shedding. Further exploration of the mechanism underlying the rebound in SARS-CoV-2 in this population will be crucial for preventing virus spread and developing effective vaccines.

## Introduction

Severe acute respiratory syndrome coronavirus 2 (SARS-CoV-2), the pathogen causing coronavirus disease 2019 (COVID-19), differs in many aspects from severe acute respiratory syndrome coronavirus (SARS-CoV) and Middle East respiratory syndrome coronavirus (MERS-CoV), which are in the same genus, Betacoronavirus. SARS-CoV-2 is relatively more infectious^1^ and infected nearly 18 million people by July 31, 2020. The virus is transmissible before the onset of clinical symptoms.^2^ SARS-CoV-2 infection is less fatal than SARS-CoV and MERS-CoV infections, but it causes a broad spectrum of clinical manifestations; in terms of severity, COVID-19 ranges from asymptomatic to mild and moderate to severe and critical.^3,4^ Notably, some discharged COVID-19 patients have positive retest results for SARS-CoV-2 RNA during follow-up,^5–7^ which increases the complexity of the disease. This causes public health concerns, such as the origin of the virus in such patients, whether the virus is transmissible, and which patients will have positive retest results. Positive detection of SARS-CoV-2 in discharged patients during follow-up is usually regarded as a recurrence of the original virus after the epidemiological exclusion of a new infection^6,8–11^ based on the fact that the discharged patients underwent a 14-day home quarantine according to the Chinese government treatment guidelines. However, this postulation has seldom been experimentally supported. As the number of discharged patients increases, their effective management becomes critical to successfully curbing the spread of the virus.

SARS-CoV-2 neutralizing antibodies are essential for viral control. Antibody generation in COVID-19 patients seems to differ significantly among individuals.^12,13^ The ability of patients who have positive retest results to produce a protective immune response might be compromised; this issue has not been extensively investigated. Currently, the factors contributing to viral recurrence are poorly understood. Elucidating the mechanism underlying viral recurrence will benefit not only patient management but also the development of effective therapies and vaccines that are suitable for the whole population.

## Results

### The gastrointestinal tract was the primary site of SARS-CoV-2 recurrence

Two hundred eighty-nine COVID-19 patients with complete disease records and at least one follow-up viral RNA test were enrolled. Throat swabs were collected for viral RNA detection by the local CDC, and the viral RNA-positive retest patients were transferred to our hospital for another round of treatment. We double-checked the viral RNA detection results in the throat, anal and sputum (rare) samples. In total, 21 patients (7.3%) were hospitalized two or more times due to testing positive for SARS-CoV-2 RNA after the initial discharge. Because of the clinical specialty of virus recurrence, both throat and anal samples were collected for viral RNA detection from all the positive retest patients during their second hospitalization. Patients who did and did not have positive retest results were designated the positive retest group and the negative retest group, respectively. Among the 268 negative retest patients, 157 patients (58.6%) had measurable viral RNA during their initial hospitalization, and 111 patients had undetectable viral RNA throughout their initial hospitalization.

**Fig. 1:**
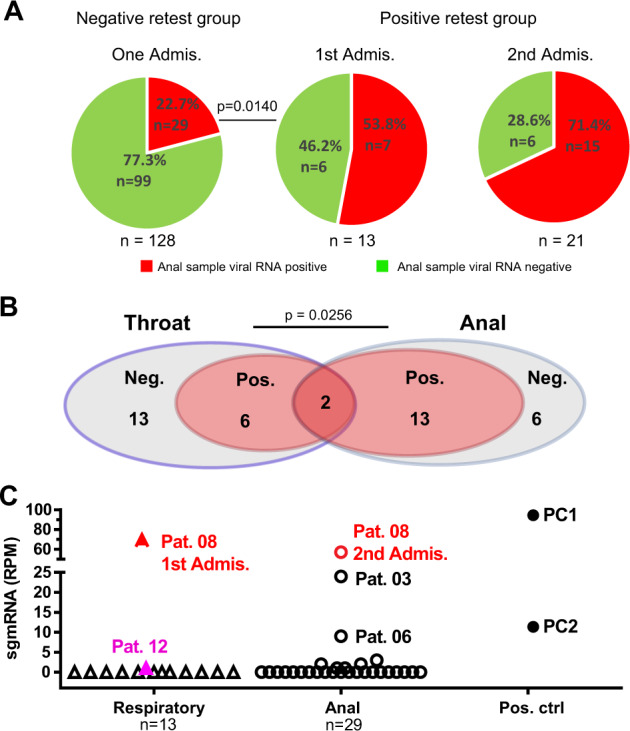
Existence of active replicating SARS-CoV-2 in anal samples from positive retest patients. **a** Frequency of viral RNA detection in anal samples between the negative retest and positive retest groups. The percentage (%) and number (*n*) are labeled. The total case number (*n*) is shown under the pie chart. *p* Values (chi-square test) are indicated. **b** Viral detection in positive retest patients during the second admission. Throat and anal samples are shown. Neg. negative samples, Pos. positive samples. **c** sgmRNA reads in samples from positive retest patients. The read numbers were normalized to reads per million (RPM) to minimize sequencing size variation. Patient numbers are shown. The positive controls were two intracellular nucleic acid samples extracted from cells with actively replicating SARS-CoV-2 (dilution factor PC1: 1 × 10^−4^, PC2: 1 × 10^−5^). Red triangle, throat sample from Patient 08 during the first admission; red circle, anal sample from Patient 08 during the second admission; pink triangle, throat sample from Patient 12 during the second admission

Interestingly, we observed that more anal samples than respiratory tract samples were positive in viral RNA-positive retest patients (Fig. 1a, Supplementary Tables 1 and 2). Among the 128 negative retest patients with anal swab samples, only 29 patients (22.7%) had detectable viral RNA in their anal samples. However, a significantly higher proportion of the positive retest patients had detectable viral RNA in their anal samples during their first admission (7 of 13, 53.8%, *p* = 0.014). Over 70% of the positive retest patients (15 of 21 patients) had detectable viral RNA in their anal swab samples during their second admission. Further analysis revealed that (1) viral RNA was more often detected in anal swabs than in throat samples (13 vs. 6 of 21 patients) and that (2) only two patients tested positive for viral RNA in both their anal swabs and throat samples (Fig. 1b, Supp;ementary Table 2). The results indicated that SARS-CoV-2 was mainly detected in the gastrointestinal tract in the positive retest patients. We postulated that the gastrointestinal tract might be one of the primary sites of virus replication during the positive retest stage.

### Active SARS-CoV-2 viral replication in the gastrointestinal tract

Since strict home quarantine measures precluded the possibility of a new infection, the virus detected in the positive retest was epidemiologically postulated to have been derived from the initial virus infection.^6,8–11^ However, experimental evidence directly supporting that conclusion has been lacking. We sequenced the viruses obtained from 42 throat and anal samples from 16 patients. Because of the extremely low viral concentrations (Supplementary Table 3), a multiplex polymerase chain reaction (PCR) amplicon-based sequencing method was used to improve the detection sensitivity. We successfully obtained the full-length SARS-CoV-2 genome (>99% genome coverage, depth ≥100-fold) from 3 (out of 16, 18.8%) patients. Fortunately, one patient (No. 08) had full-length viral genome sequences from his first admission and his second admission 35 days after discharge. Phylogenetic analysis of 65 SARS-CoV-2 genomes obtained in our hospital revealed that the virus detected during the second admission (anal swab) was closely related to the parent virus detected during the first admission (throat swab) (Supplementary Fig. 1). Therefore, we experimentally confirmed, for the first time, that the virus detected in the positive retest originated from the virus that caused the initial infection.

Unfortunately, virus isolation from these samples was impossible because of the heat inactivation that was necessary for clinical viral detection purposes. Therefore, we employed a well-accepted method that detects coronavirus sgmRNA to determine the presence of live and transmissible viruses.^14–16^ SARS-CoV-2 generates a large number of spliced sgmRNAs that contain the 5′ UTR and gene body to enable efficient viral protein production. Since sgmRNAs are only produced intracellularly in virus-infected cells and are not packaged into viral particles, their presence implies active viral replication and production. Among our sequenced samples, high concentrations of sgmRNA were detected in several anal samples from Patients 08, 03, and 06, while one respiratory sample from Patient 12 (pink triangle) during the second admission had barely detectable levels of sgmRNA in contrast to the respiratory sample from Patient 8 during the first admission (red triangle) (Fig. 1c). The sgmRNA containing the N gene was the most abundant mRNA transcript in isolated replicating SARS-CoV-2. To verify the presence of the sgmRNA containing the N gene,^15,16^ we designed specific sgmRNA primers and detected N-containing amplicons from Patients 03 and 08 (Supplementary Fig. 2A). The PCR product was further confirmed to contain the 5′ UTR and the N gene by Sanger sequencing (Supplementary Fig. 2B). The only throat sample (from Patient 12, purple triangle in Fig. 1c), which had the highest viral concentration and over 90% genome coverage, had barely detectable total sgmRNA (RPM = 1) (Supplementary Table 3) and undetectable N-containing sgmRNA (Supplementary Fig. 2A), implying the lack of robust active viral replication, if any. Interestingly, the finding that all the samples with active viral replication were anal swabs and not respiratory tract samples suggests that the gastrointestinal tract might be the site of SARS-CoV-2 persistence and shedding.

Collectively, our results supported that SARS-CoV-2 could hide and replicate at lower levels mainly in the gastrointestinal tract for a long time and that periodic viral shedding would lead to viral retest, mainly in anal samples.

### Viral titer and disease severity were not related to SARS-CoV-2 recurrence

**Fig. 2:**
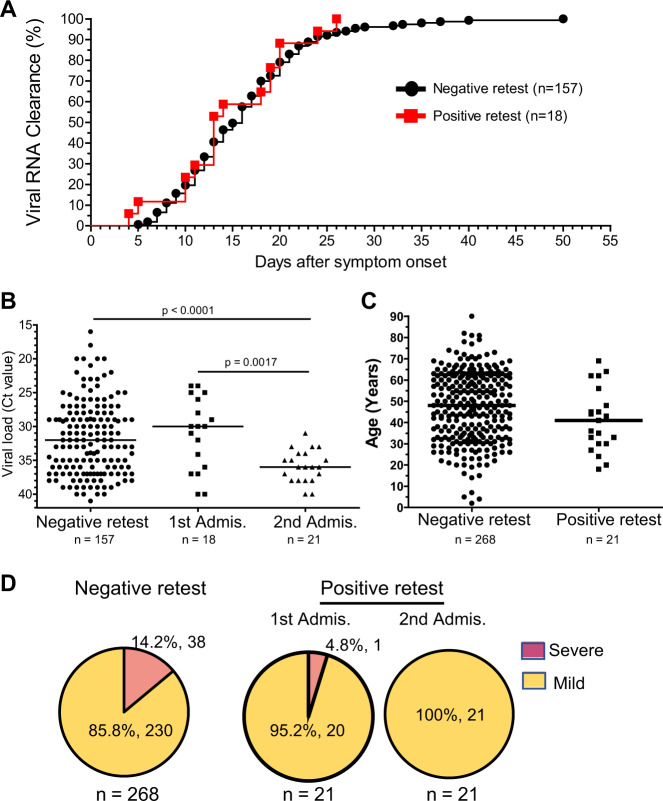
Clinical features of SARS-CoV-2 RNA-positive patients. **a** Kinetics of SARS-CoV-2 RNA clearance. The cumulative viral clearance (percentage, %) is shown for the negative retest (black circle) and positive retest groups (red square). *p* Values (log-rank (Mantel–Cox) test) are indicated. **b** Maximum viral concentration distribution. Each data point represents the maximum viral concentration (Ct value) in each patient during the entire admission. Patient numbers are labeled below each group. **c** Age distribution. An unpaired *t* test with Welch’s correction was used. *p* Values with a significant difference are shown. **d** Clinical symptom severity. Percentage (%) and number (*n*) are indicated

We suspected that factors such as high viral titers and severe clinical manifestations might cause the virus to rebound. First, we adopted viral RNA clearance as the end event instead of patient discharge, which is a complex endpoint that incorporates more subjective and nonviral factors. Viral RNA clearance was defined as the time at which the second consecutive negative RNA test result was obtained, using either throat samples or anal samples, although the latter were not routinely measured. Unexpectedly, 90% of the patients from the negative retest group, who only had one hospitalization (*n* = 268 patients), and the positive retest group during their first hospitalization (*n* = 21 patients) achieved viral clearance within 23–24 days (Fig. 2a). The time to achieve complete viral clearance in the positive retest group (90% of the individuals) from the initial symptom onset was 48–57 days (*n* = 21 patients) (data not shown).

Second, we analyzed the peak viral titers during the first admission. Since every patient had multiple viral RNA tests during hospitalization, we chose the highest viral RNA titers (the lowest Ct values) as the representative viral concentration (Fig. 2b). Interestingly, no difference was observed between the negative retest group (*n* = 157) and the positive retest group (*n* = 18). However, we found a significantly lower viral concentration in the positive retest patients during their second admission (*n* = 21, *p* < 0.0001 vs. negative retest group, *p* = 0.0017 vs. the positive retest group during their first admission).

Third, age is an essential factor that affects the clinical outcomes of SARS-CoV-2 infection.^17,18^ Regarding the patient age distribution, no significant difference was observed between the negative retest group (*n* = 268) and the positive retest group (*n* = 21) (Fig. 2c). In addition, the sex distributions were similar between these two groups (data not shown).

Finally, we suspected that viral persistence was due to disease severity. A comprehensive analysis of the clinical manifestations revealed that the positive retest group had a nonsignificantly smaller proportion (4.8%) of severe cases during the first hospitalization than the negative retest group (14.2%) (*p* > 0.05, Fig. 2d). In addition, all patients had mild symptoms during their second hospitalization (Fig. 2d).

Collectively, these observations demonstrated that viral rebound was not related to age, gender, or the rate of viral clearance. Although not reaching a statistically significant difference, there was a tendency of higher viral concentration and more mild cases in positive retest patients than in negative retest patients during their first hospitalization.

### Compromised anti-RBD-specific antibody generation in positive retest patients

**Fig. 3:**
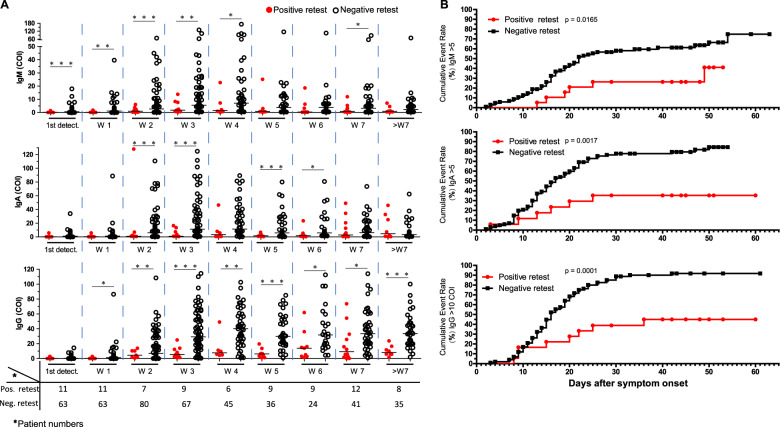
Features of anti-RBD-specific IgM, IgA, and IgG. **a** Concentrations (cut-off index, COI) of anti-RBD-specific IgM (upper), IgA (middle), and IgG (bottom) antibodies at different time points. Times (weeks after symptom onset, W) are as marked. First, serum detection for each patient within 1 week after symptom onset was grouped separately as “1st detect.” Patient numbers at each timepoint are labeled separately for the positive retest group (red filled circle, positive retest.) and the negative retest group (black open circle, negative retest.). An unpaired *t* test with Welch’s correction was used. *p* Value: *p < 0.05, **p < 0.01, ***p < 0.001. **b** The speed of anti-RBD-specific antibody generation. Cumulative patient numbers (%) with anti-RBD IgM > 5 COI (upper), IgA > 5 COI (middle), and IgG > 10 (bottom) are shown. Positive retest group, red filled circle; negative retest group, black filled square. *p* Values (calculated by the log-rank (Mantel–Cox) test) are shown

We then suspected that positive retest patients might fail to generate protective antibodies. Using a sensitive immunodetection assay that precisely measures spike protein receptor-binding domain-specific antibodies,^19^ we measured the serum anti-RBD IgM, IgA, and IgM levels at different stages. Compared to the negative retest patients (*n* = 158), the positive retest patients (*n* = 21) had significantly lower levels of IgM, IgA, and IgG (Fig. 3a). In the negative retest group, high concentrations of IgM were detected within 2 weeks; these levels increased and were maintained until 4 weeks after illness onset and then declined (Fig. 3a, upper). In the positive retest group, IgM was weakly elicited in a few patients. Similarly, the serum IgA levels started to increase within 2 weeks and were maintained until week 7 in the negative retest patients. The positive retest patients showed delayed IgA generation until week 7 (Fig. 3a, middle). Importantly, the IgG level reached a plateau within 3 weeks, with a median value >20 cut-off index (COI). The positive retest individuals generated significantly lower levels of RBD-specific IgM, IgA, and IgG antibodies throughout the disease course.

Then, we characterized the dynamic changes in antibodies. A cut-off value of COI = 5 was used for IgM and IgA, and COI = 10 was used for IgG (Fig. 3b). More than 70% of the negative retest patients obtained a high level of IgM, but less than 45% of the positive retest patients produced a high level of IgM (Fig. 3b upper, *p* = 0.0165). More negative retest patients than positive retest patients generated a high level of IgA (85% vs. 35% *p* = 0.0017, Fig. 3b middle). More negative retest patients than positive retest patients generated a high level of IgG (91% vs. 45% *p* = 0.0001, Fig. 3b bottom).

We then measured the neutralization potential of the serum from representative patients at multiple time points using a SARS-CoV-2 cell infection system and found that the levels of IgG and IgA, but not IgM, were well correlated with the serum microneutralization titers (Supplementary Fig. 3), supporting the idea that the levels of anti-RBD IgG and IgA could represent the serum neutralization capacity.

In short, our results imply that positive retest individuals might fail to generate protective antibodies in a timely manner, resulting in delayed viral clearance.

### Diverse clinical outcomes of COVID-19 patients

**Fig. 4:**
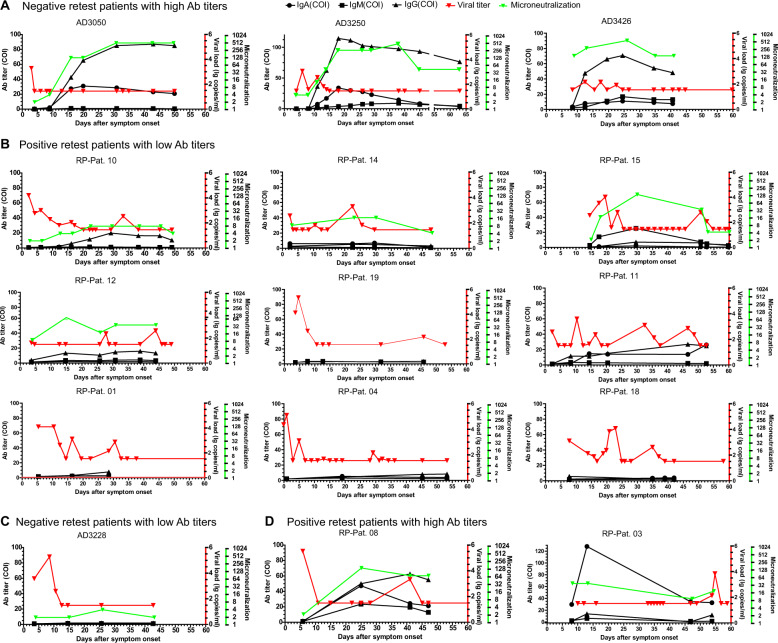
Kinetics of viral RNA, anti-RBD antibodies, and neutralizing capacity. **a** Profiles of three representative negative retest COVID-19 patients with high levels of anti-RBD antibodies. Fifty-eight of 60 negative retest patients were in this group (see Supplementary Fig. 4). **b** Profiles of nine representative positive retest COVID-19 patients with low levels of anti-RBD antibodies. Seventeen of 19 positive retest patients were included in this group. **c** Profiles of one negative retest patient with low anti-RBD antibody titers and nonprotective neutralizing activity. Two of 60 negative retest patients were in this group. **d** Profiles of two positive retest patients with high levels of antibodies and neutralizing activity. Two of 19 positive retest patients were in this group. Black lines, IgM, IgA, and IgG; red line, viral load; green line, serum microneutralization. Patient ID numbers are shown on the top. Viral RNA below the detection limit was set at 1.44 log10. Folds of serum dilution were used as microneutralization titers

COVID-19 patients have varying clinical symptoms. Here, most negative retest individuals (58 of 60, 96.7%) generated high titers of viral RBD-specific IgG or/and IgA antibodies (Fig. 4a, Supplementary Fig. 4), and they produced more protective antibodies (>1:128 dilution). Most positive retest individuals (17 of 19, 89.5%) produced low titers of RBD-specific antibodies, and their neutralization capacity was compromised (Fig. 4b). Interestingly, 2 (out of 60, 3.3%) negative retest patients could efficiently and effectively control the virus but had low concentrations of RBD-specific IgA and IgG antibodies, and their serum was unable to be infected by live viruses in the cell culture system (Fig. 4c), which suggests that nonhumoral immunity contributes to viral control. In addition, 2 of 19 positive retest patients generated high levels of RBD-specific IgA (>40 for RP-Pat. 08, >120 COI for RP-Pat. 03) and IgG (>60 COI for RP-Pat. 03) antibodies, which had neutralization capacity with regard to preventing the infection of cells by live SARS-CoV-2 viruses (≥1:32 dilution). Nevertheless, they failed to suppress viral replication completely (Fig. 4d). In summary, our observations demonstrated that patients have a broad spectrum of clinical manifestations and suggest that multiple branches of the immune response participate in SARS-CoV-2 control.

## Discussion

In this study, we demonstrated that the gastrointestinal tract is the main extrarespiratory site of SARS-CoV-2 persistence and periodic viral shedding. The digestive tract, which expresses a high level of angiotensin-converting enzyme 2 (ACE2), the SARS-COV-2-binding receptor,^20^ is a site of efficient SARS-COV-2 viral infection.^21,22^ First, we observed that the anal samples from the positive retest patients were more often positive for viral RNA than the anal samples from negative retest patients during the first hospitalization (53.8 vs. 22.7%, Fig. 1a) and that more than 70% of positive retest patients had detectable viral RNA in their anal samples during their second hospitalization. Second, active SARS-CoV-2 viral replication was observed in the anal samples but not in the respiratory samples. The viral titer was lower in the positive retest stage (second admission, Fig. 2b), which prevented direct virus isolation. We managed to obtain a nearly full-length viral genome sequence in 3 of 16 positive retest patients (Fig. 1b) using an improved viral RNA enrichment sequencing method^23^ with markedly increased depth. The presence of viral sgmRNA is widely accepted as direct evidence of the replication of coronaviruses.^14,24^ sgmRNA detection has been used as an alternative method of analyzing the infectivity of SARS-CoV-2.^15,16^ In our analysis, the sgmRNA concentrations of two positive controls extracted from actively replicating SARS-CoV-2 cell cultures with a 10-fold difference were 96 and 12 reads per million (RPM), respectively, and the RPM of sgmRNA from 3 of 16 positive retest patients (Patients 08, 03, and 06) were 57, 24, and 9, respectively. Therefore, our data indicated that active viral replication occurred in the gastrointestinal tract, suggesting the potential production of infectious progeny viruses. Collectively, our results indicated that the gastrointestinal tract could be a vital reservoir of low levels of SARS-CoV-2 replication, which might lead to the periodic shedding of potentially infectious viruses. This finding highlights the necessity of stringent public hygiene measures to prevent viral transmission by positive retest patients.

Our longitudinal investigation of the RBD-specific antibody titers from disease onset to complete cure suggested that neutralizing antibodies usually play critical roles in viral control in COVID-19 patients. The viral concentration (Fig. 2b) and speed of viral RNA clearance (Fig. 2a) were mainly measured in the upper respiratory tract, and the illness severity during the first hospitalization was not related to viral rebound (Fig. 2d). Neutralizing antibodies play critical roles in viral control by limiting new infections and neutralizing free viruses. Antibodies specifically targeting the RBD of the SARS-CoV-2 spike protein can effectively prohibit viral entry into the cell. The antibody concentrations can reflect the capacity of the host to prevent viral infection.^25^ Our RBD-specific antibody profiling analysis revealed that the positive retest patients generated lower titers of IgM, IgA, and IgG antibodies and generated them more slowly (Fig. 3). The subsequent serum microneutralization assay also confirmed that the representative positive retest patients failed to generate potent and long-lasting neutralizing antibodies, unlike the negative retest patients (Fig. 4, Supplementary Fig. 4).

In addition, we experimentally confirmed that viral recurrence prolongs viral shedding from the reservoir in the gastrointestinal tract. Phylogenetic analysis of the full-length SARS-CoV-2 genome in the positive retest stage demonstrated that the virus detected in the positive retest had evolved from the parent virus involved in the initial infection. To our knowledge, this is the first experimental confirmation of the origin of the virus detected in the positive retest. Unfortunately, we only analyzed paired virus genomes from one representative patient in this report. We are still following the clinical cohort to collect samples from additional patients to perform a further analysis of positive retest patients. One recent study reported detectable SARS-CoV-2 in the lung biopsy of a woman who was nearly discharged after negative RNA tests of throat swabs, which suggested the possibility of viral rebound due to persistence of the virus.^26^

Our study has some limitations. First, as this was a retrospective analysis, sample collection did not follow a stringent timeline. Therefore, most of the patients had missing time points, especially in the early stage when the presence of the virus in the gastrointestinal tract was ignored. Second, because all clinically collected throat and anal swab samples were inactivated, it was impossible to perform viral isolation or to conduct additional experiments using live viruses. Last, the specific cellular response to SARS-CoV-2, which might be vital to eventually achieving viral control in the absence of a potent humoral response in positive retest patients, was unavailable.

In summary, our analysis demonstrated that positive retest patients had delays in the development of RBD-specific IgA and IgG and eventually developed relatively low levels of these antibodies; these factors may have contributed to viral persistence in the gastrointestinal tract and the subsequent periodic viral shedding and delayed viral clearance. Further identification of the fundamental mechanisms resulting in a compromised humoral immune response will benefit the development of effective vaccines suitable for the protection of the entire population.

## Methods

### Patients

Two hundred ninety-seven patients admitted to Guangzhou Eighth People’s Hospital were enrolled in this retrospective study. General patient information, including age, sex, and clinical diagnosis, was collected from the hospital information system. All patients were diagnosed based on their clinical manifestations according to the Guidelines for the Diagnosis and Treatment of Novel Coronavirus Infection produced by the Chinese National Health Commission (Trial Version 7). The study was approved by the medical ethics committee of Guangzhou Eighth People’s Hospital (No. 202001134). Written consent was obtained from all patients.

### Measurement of RBD-specific IgA, IgG, and IgM antibodies

Plasma samples were inactivated at 56 °C for 30 min and stored at −80 °C before testing. IgA, IgG, and IgM antibodies against the SARS-CoV-2 RBD spike protein in plasma samples were tested with two-step indirect immunoassay electrochemiluminescence immunoassay kits as previously reported^19^ (Kangrun Biotech Co., Ltd.), according to the manufacturer’s instructions. Briefly, the samples were first incubated with microparticles coated with the RBD of the SARS-CoV-2 spike protein and acridine ester-labeled antibodies against the Fc domain of human antibodies. After the unbound substances were washed off, signal detection was performed on an automatic chemiluminescence immunoanalyzer (KAESER1000, Chongqing Cosmax Biotech Co., Ltd.). All tests were performed under strict biosafety conditions.

### Viral RNA detection by RT-PCR

Viral RNA was extracted using a Nucleic Acid Isolation Kit (Da’an Gene Corporation, Cat: DA0630) on an automatic workstation Smart 32 (Da’an Gene Corporation) according to the instructions. Real-time reverse transcription polymerase chain reaction (RT-PCR) reagent (Da’an Gene cooperation, Cat: DA0930), which targets the N and orf1ab genes, was employed for viral detection per the protocol. A Ct value <40 was regarded as a positive result. Viral DNA standards were used as references to calculate the viral RNA concentration.

### Viral RNA sequencing and analysis

The sequencing library was prepared using an amplicon-based enrichment method as described previously,^23^ except that the cycle numbers of the first and second rounds of PCR were modified to 13 and 27, respectively. The sequencing library of all the other samples was prepared using the hybrid capture-based enrichment method as previously reported. All the samples were sequenced on the MGISEQ-2000 platform. Genomic assembly was conducted using the nCoV Finder pipeline (https://github.com/BGI-IORI/nCoV_Meta). Variation detection was carried out using the nCoV Variant detection pipeline (BGI-Shenzhen) for hybrid capture-based sequencing data (https://github.com/BGI-IORI/nCoV_Variants) and SARS-CoV-2 Multi-PCR v1.0 (MGI Tech Ltd., Co.) for amplicon-based sequencing data (https://github.com/MGI-tech-bioinformatics/SARS-CoV-2_Multi-PCR_v1.0). To guarantee reliable variation detection in positive retest samples, only mutation sites with a sequencing depth greater than 100× were reported.

sgmRNA detection was performed for sequencing reads from samples from positive retest patients. Clean reads were obtained after the removal of low-quality reads and adaptors and primer trimming. Reads were then aligned to the SARS-CoV-2 reference genome (NC_045512) with HISAT2,^27^ and the junction sites were extracted using the RegTools junctions extract command.^28^ Statistical analysis of the read numbers for SARS-CoV-2 was performed with SAMtools.^29^

### Phylogenetic tree construction

The genomes were aligned using MAFFT v7.427 6 and manually checked with BioEdit. Phylogenetic trees were generated based on full genomes using the maximum likelihood (ML) method implemented in the program IQ-TREE v1.6.12 with the best-fit model for nucleotide substitution determined by ModelFinder.^30^ Bootstrap support values were calculated from 1000 pseudoreplicate trees. Visualization of the phylogenetic tree was conducted with the ggtree package.^31^

### Microneutralization assay

Heat-inactivated serum was serially diluted 4-fold (from 1:4 to 1:1024) and then mixed with an equal volume (125 μl) of a viral solution containing 100 TCID50 of SARS-CoV-2. Next, serum-virus mixtures were first incubated for 2 h at 37 °C and were then applied to a semiconfluent VERO E6 monolayer in duplicate. After a 4-day incubation, virus-infected wells were assessed.

### Statistical analysis

The log-rank (Mantel–Cox) test, unpaired *t* test with Welch’s correction, and chi-square test were used to analyze the data in GraphPad PRISM software (Version 5.01).

## Supplementary information

Table S1

Table S2

Table S3

Fig S1

Fig S2

Fig S3

Fig S4

## Data Availability

Sequence data that support the findings of this study have been deposited into CNSA (China National GeneBank Nucleotide Sequence Archive, https://db.cngb.org/cnsa/) under project numbers CNP0000944 and CNP0001099.
