# Supplementary figures and images for: A compromised specific humoral immune response against the SARS-CoV-2 receptor-binding domain is related to viral persistence and periodic shedding in the gastrointestinal tract
Source: Cell Mol Immunol. 2020 Oct 9;17(11):1119–25. doi: 10.1038/s41423-020-00550-2 (PMC7546387; doi:10.1038/s41423-020-00550-2)

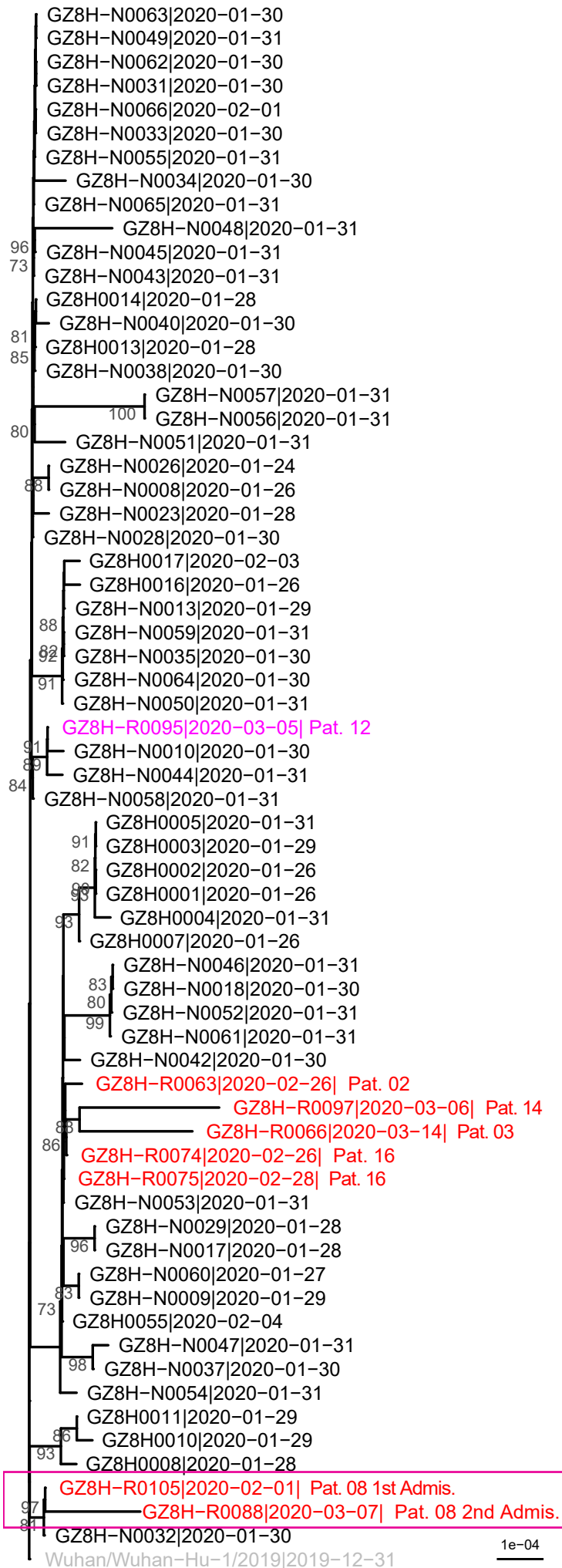

Supplement: Supplementary file 4 — Fig S1 [file 41423_2020_550_MOESM4_ESM.pdf]

A

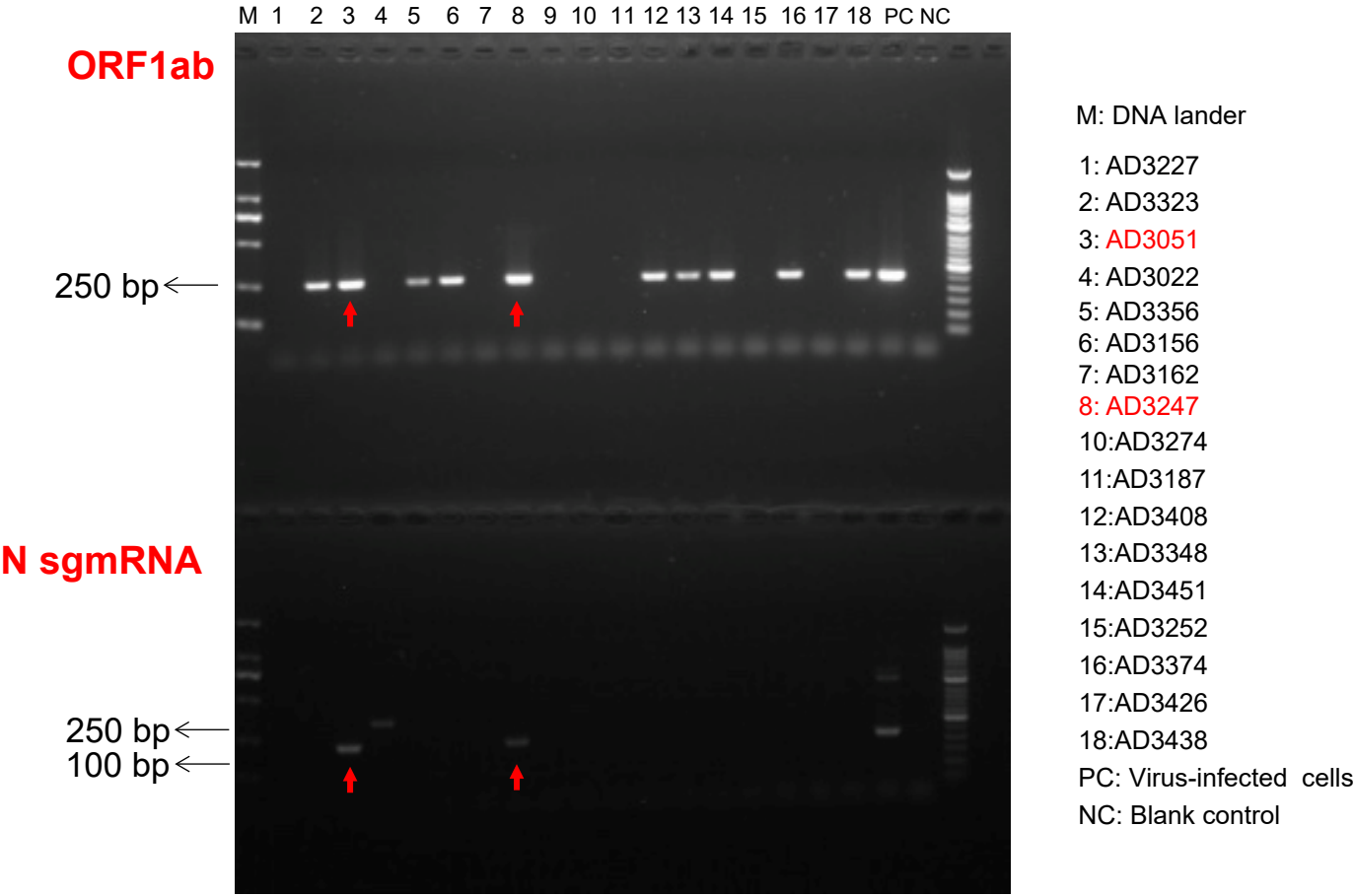

B

Patient\_03: AD3051

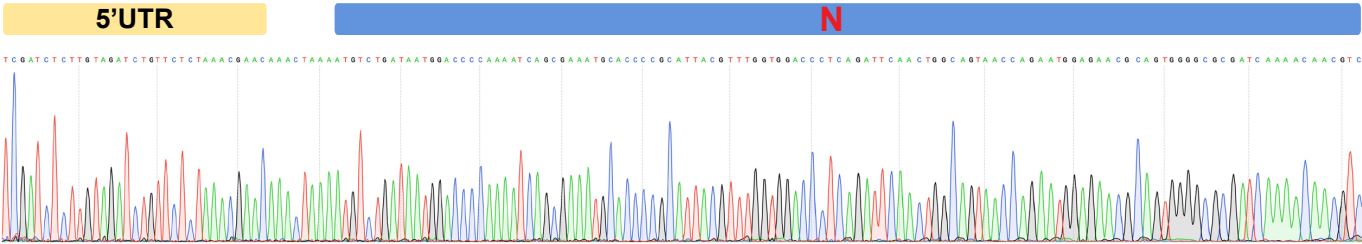

Patient\_08: AD3247

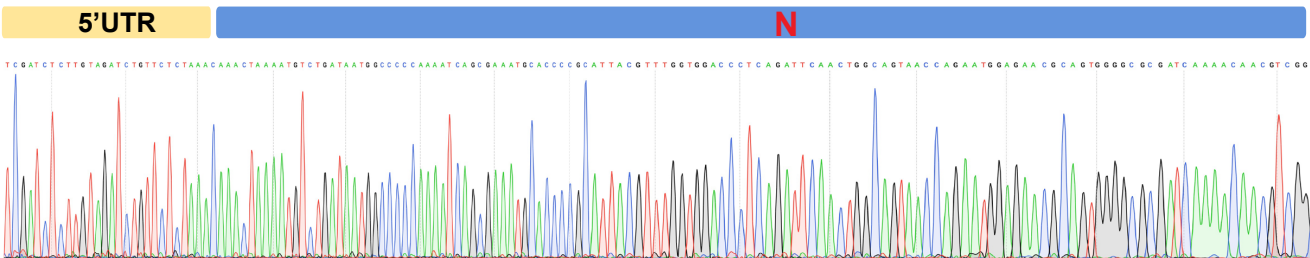

Supplement: Supplementary file 5 — Fig S2 [file 41423_2020_550_MOESM5_ESM.pdf]

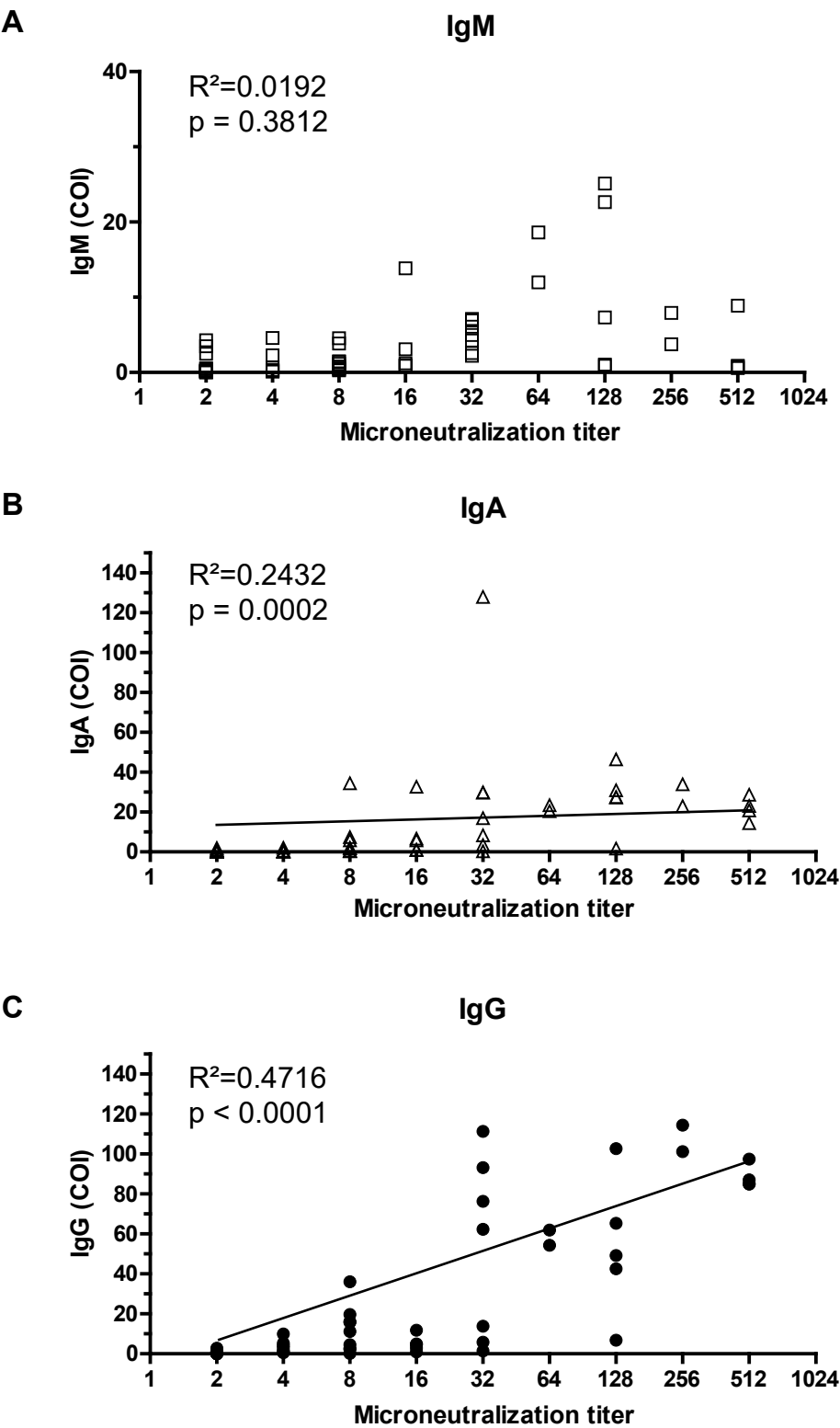

Supplement: Supplementary file 6 — Fig S3 [file 41423_2020_550_MOESM6_ESM.pdf]

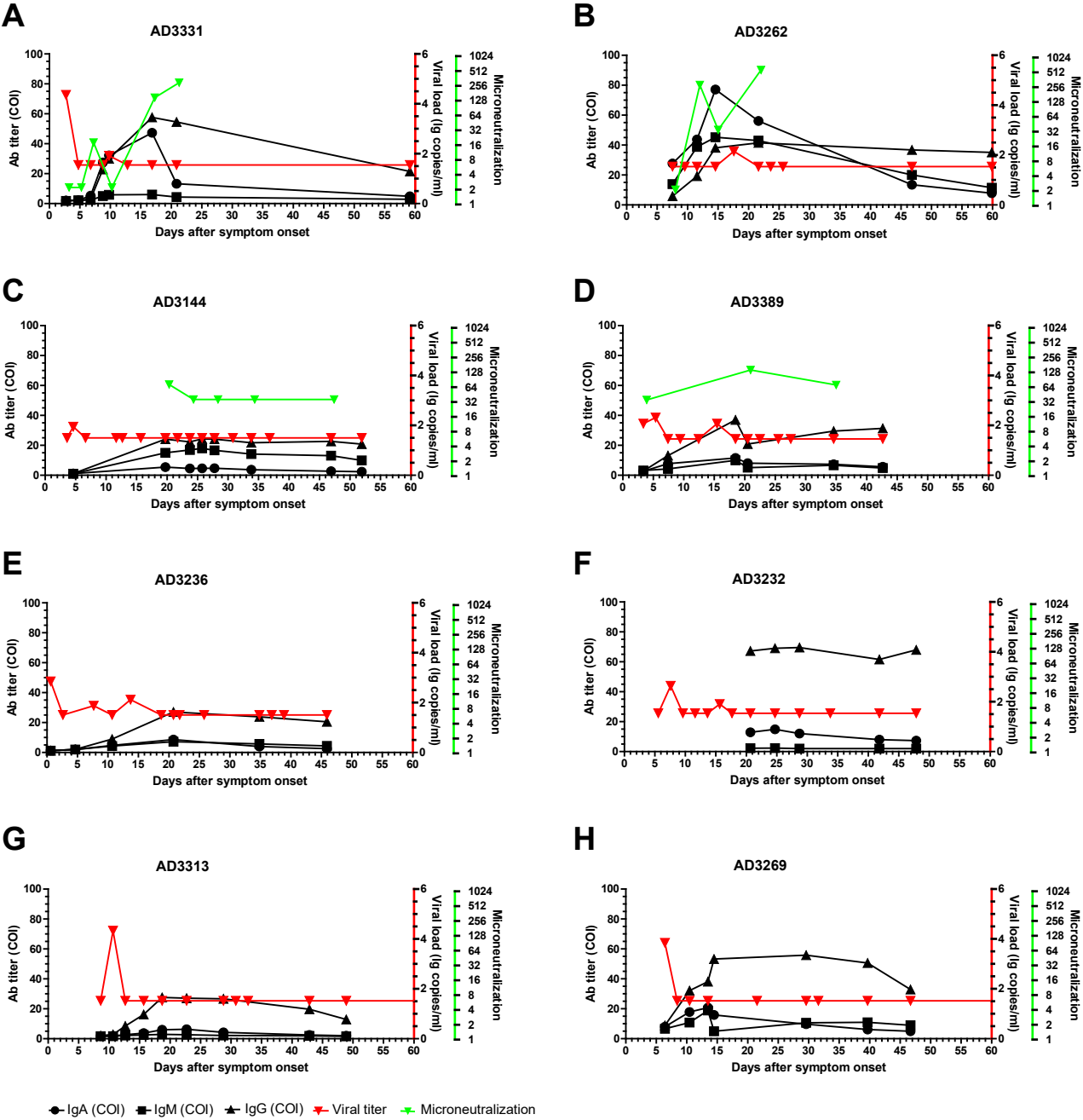

Supplement: Supplementary file 7 — Fig S4 [file 41423_2020_550_MOESM7_ESM.pdf]
